# Papillary renal cell carcinoma-derived chemerin, IL-8, and CXCL16 promote monocyte recruitment and differentiation into foam-cell macrophages

**DOI:** 10.1038/labinvest.2017.78

**Published:** 2017-07-31

**Authors:** Krzysztof M Krawczyk, Helén Nilsson, Roni Allaoui, David Lindgren, Michael Arvidsson, Karin Leandersson, Martin E Johansson

**Affiliations:** 1 Department of Translational Medicine, Center for Molecular Pathology, Lund University, Malmö, Sweden; 2 Department of Translational Medicine, Cancer Immunology, Lund University, Malmö, Sweden; 3 Department of Laboratory Medicine, Translational Cancer Research, Lund University, Lund, Sweden

## Abstract

Papillary renal cell carcinoma (pRCC) is the second most common type of renal cell carcinoma. The only curative treatment available for pRCC is radical surgery. If the disease becomes widespread, neither chemo- nor radiotherapy will have therapeutic effect, hence further research on pRCC is of utmost importance. Histologically, pRCC is characterized by a papillary growth pattern with focal aggregation of macrophages of the foam cell phenotype. In other forms of cancer, a clear role for tumor-associated macrophages during cancer growth and progression has been shown. Although the presence of foamy macrophages is a histological hallmark of pRCC tumors, little is known regarding their role in pRCC biology. In order to study the interaction between pRCC tumor and myeloid cells, we established primary cultures from pRCC tissue. We show that human pRCC cells secrete the chemokines IL-8, CXCL16, and chemerin, and that these factors attract primary human monocytes *in vitro*. RNAseq data from The Cancer Genome Atlas confirmed a high expression of these factors in pRCC tissue. Conditioned medium from pRCC cultures induced a shift in human monocytes toward the M2 macrophage phenotype. In extended cultures, these macrophages became enlarged and loaded with lipids, adopting the foam cell morphology found in pRCC tissue. These results show for the first time that pRCC primary tumor cells secrete factors that attract and differentiate monocytes into anti-inflammatory tumor-associated macrophages with foam cell histology.

Papillary renal cell carcinoma (pRCC) accounts for 15–20% of all renal cell carcinoma (RCC) cases, which makes it the second most common subtype.^1^ Like for other types of RCC, radical surgery is the only curative treatment option for pRCC. If surgery fails or metastatic disease is present at diagnosis, neither radio- nor chemotherapy has therapeutic effect. Targeted therapies like sunitinib or temsirolimus can offer prolonged survival for patients with the clear cell subtype of RCC. Unfortunately, only limited effects are seen on papillary RCC. Histologically, pRCC architecture is characterized by a predominantly papillary growth pattern, where the fibrovascular stalks are covered by the malignant cells. In a minority of cases, tubular and sometimes solid patterns may also occur in parts of the tumor. Based on cytology and morphology, pRCC has been subdivided into type 1 and type 2, where type 2 entails worse prognosis. Type 1 pRCC is characterized by papillae covered by comparatively small cancer cells, often with amphophilic cytoplasm and low nuclear grade. Type 2 pRCC denotes papillae covered by larger eosinophilic cells with more prominent nuclei and nuclear pseudostratification.^2, 3^ This division has recently gained support from molecular genetic profiling of mutations, copy number, and methylation patterns of DNA together with RNA expression and proteomic analysis of pRCC cases deposited in The Cancer Genome Atlas (TCGA) cohort.^1, 4^

A histological hallmark found in a majority of pRCC cases is focal accumulation of foam cell macrophages in the stroma of the papillary stalks.^2^ Foam cells are generally regarded as lipid-laden macrophages, as for instance seen in atherosclerotic plaques or gall bladder cholesterolosis. Whether this is also true for the pRCC-associated macrophages is not established. Interestingly, the foam cell morphology of the macrophages present in pRCC is not seen in any other cancer form, and very little is known regarding their development and tumor biological impact. The presence of tumor-associated macrophages (TAMs) in different forms of cancer has during recent years attracted considerable attention, as they may elicit both pro- and anti-tumoral responses. In this context, they can be divided into classical M1 and alternatively activated M2 forms. The M1 form is regarded as inflammatory, cytotoxic, and anti-tumoral, whereas the M2 form has anti-inflammatory, immune-suppressive and wound healing properties, that may support neoplastic growth.^5, 6^

Despite being a characteristic histological hallmark, few studies have addressed the mechanisms behind the attraction and retention of macrophages in pRCC tumors. Little is known regarding the factors that attract monocytes, the precursors of macrophages, into these tumors, or how these cells affect the tumor behavior and microenvironment. One reason may be the lack of relevant *in vitro* models, as there are no cell lines with confirmed pRCC origin, and successful primary culture of pRCC is difficult and therefore rarely used.

In this study, we have successfully cultured primary human pRCC cells isolated from patient nephrectomies, and investigated their interaction with primary human myeloid cells. We find that cultured pRCC cells secrete the chemokines CXCL16, IL-8, and the adipokine chemerin (RARRES2) into their surroundings. Both IL-8 and CXCL16 have previously been shown to attract monocytes in a tumor microenvironment.^7, 8^ Chemerin expression at mRNA level has been reported from malignancies such as mesothelioma^9^ and adrenocortical tumors.^10^ However, the role of chemerin in tumor biology remains unclear. We show that CXCL16, IL-8, and chemerin, alone or in combination, as well as conditioned medium from pRCC cultures, attract human primary monocytes. Also, extended culture of monocytes in pRCC-conditioned medium induces differentiation toward an M2-like, lipid-laden foam cell phenotype, closely mimicking the macrophages found in pRCC tumor tissue samples.

These findings indicate that pRCC cells actively recruit myeloid cells and form the unique foam-cell inducing tumor microenvironment of pRCC. Indeed, based on our results, the tumor microenvironment and the factors produced by pRCCs seem to differ from most conventional tumors studied previously.

## MATERIALS AND METHODS

### Primary Cell Culture

pRCC specimens were obtained after patients written informed consent from nephrectomies performed at Skåne University Hospital (Malmö, Sweden) with ethical approval from Lund University ethical committee (LU680-08 and LU 289-07). All pRCC cases used for primary cultures were diagnosed by a pathologist specialized in urological pathology according to standard clinical practice, including evaluation of immunohistochemical markers for pRCC. The diagnosis was validated by an additional urological pathologist before the material was used. Furthermore, the pRCC origin of the cultured cells was confirmed by staining for CK7, CK19, and vimentin. Tumor tissue was collected in serum-free DMEM high-glucose medium (GE Healthcare, Little Chalfont, UK) supplemented with 1% penicillin–streptomycin (GE Healthcare), transferred on ice to the cell culture facility and prepared immediately for culture. Tissue samples were cut into smaller pieces with surgical blades and digested overnight in full DMEM medium containing Collagenase Type I (300 U/ml, Thermo Fischer Scientific, Waltham, MA, USA) and DNase I, Type II (200 U/ml, Sigma Aldrich, St Louis, MO, USA). The following day samples were treated with Trypsin (GE Healthcare) for 5 min and thereafter sequentially filtered through 40 and 20 μm cell strainers to obtain single-cell solution. Isolated cells were cultured in DMEM high-glucose medium supplemented with 10% fetal bovine serum (Saveen Werner, Limhamn, Sweden) and 1% penicillin–streptomycin in 37 °C, 5% CO_2_, in a humidified incubator.

Conditioned medium was collected from subconfluent cultures after 24 h incubation in serum-free medium. The conditioned medium was spun down to remove cell debris, aliquoted and stored in −80 °C until further use.

### Patient Material

Paraffin-embedded tissue samples from patients undergoing radical or partial nephrectomy at the Department of Urology at the University Hospital in Malmö, Sweden between 1997 and 2016 were analyzed. Ethical approval was obtained from Lund University ethical committee (LU680-08 and LU 289-07). In total, 140 cases histologically confirmed as pRCC were included. All tumors were re-evaluated histopathologically and macrophage content was assessed in multiple sections from each tumor. Fuhrman and WHO/ISUP grades were determined and tumors were classified into type 1 and type 2 according to the WHO classification,^3^ with the exception that tumors containing a combination of type 1 and type 2 features were divided into a separate group. Clinical tumor data are summarized in Tables 1 and 2. A tissue micro array was prepared containing 51 of these tumors in duplicates. Clinical tumor data for these tumors are summarized in Supplementary Table 1.

### Histology and Immunohistochemistry

Tumor tissue or cultured cells were fixed in formalin and paraffin embedded according to routine protocol. Deparaffinization and rehydration of tissue sections of 4 μm thickness was performed according to standard procedures. Sections were subjected to epitope retrieval in a PT Link module (Dako, Santa Clara, CA, USA) and immunohistochemical stainings were performed using an Autostainer Plus (Dako) according to the manufacturer’s standard protocol. Antibodies used were: cytokeratin 7 (Dako, M7018, 1:100) cytokeratin 19 (Ventana, Basel, Switzerland, 760-4281, prediluted), vimentin (Ventana, 790-2917, prediluted), CD10 (Ventana, 790-4506, prediluted) CD31 (Ventana, 760-4378, prediluted), CD68 (Dako, M0814, 1:1000), CD163 (Ventana, 760-4437, prediluted), and CXCL16 (Abcam, Cambridge, UK, ab101404, 1:100). Sections were stained with hematoxylin and eosin or counterstained with hematoxylin.

### Chemokine Array

Chemokine secretion profiling of cultured primary pRCC cells was performed on pRCC-conditioned medium using a human chemokine proteome profiler antibody array (R&D Systems, Minneapolis, MN, USA) according to the manufacturer’s instructions.

### TCGA Gene Expression Data

Level 3 RNA sequencing data containing mRNA gene-level RSEM estimates were downloaded from The Cancer Genome Atlas (TCGA) data portal (http://tcga-data.nci.nih.gov/tcga/dataAccessMatrix.htm) by September 2014. Gene expression levels for each sample were created by multiplying the gene level RSEM estimate values by 10^6^ followed by adding an offset of 1 and subsequent log2 transformation. The data set comprised 234 pRCCs and 32 normal kidney tissue samples.

### Isolation of Human Monocytes

Leukocytes were prepared from leukocyte depletion filters used for healthy blood donors according to previously published methods.^11^ Further extraction of peripheral blood mononuclear cells (PBMCs) was performed within 2–3 h. PBMCs were extracted using Ficoll-Paque PLUS (GE Healthcare) gradient centrifugation and monocytes were isolated by a magnetic cell sorting procedure using Monocyte Isolation Kit II (Miltenyi Biotec, Bergisch Gladbach, Germany), according to instructions provided by the manufacturer. The antibody cocktail used in order to negatively select for monocytes was directed against CD3, CD7, CD16, CD19, CD56, CD123, and glycophorin A. Ethical permit for using blood from healthy blood donors was obtained from the regional ethical committee at Lund University (Dnr 2012/689).

### Migration Assay

Isolated monocytes were seeded in serum-free medium into the upper well of 8 μm Polycarbonate Permeable Supports (Corning Costar, Corning, NY, USA). To the lower compartment, serum-free medium, conditioned medium from primary human pRCC cell cultures, or serum free medium with addition of recombinant human CXCL16 (100 ng/ml), IL-8 (100 ng/ml, R&D Systems), chemerin (100 ng/ml, PeproTech, Rocky Hill, NJ, USA), or the mixture of all three was added. After 24 h, migrated cells were collected and monocytes were counted using flow cytometry.

### Differentiation Assay

Phenotypic changes of monocytes were analyzed after 1 week in culture with DMEM high glucose supplemented with 10% human serum (Sigma Aldrich) with or without pRCC-conditioned medium. Monocytes were harvested using non-enzymatic dissociation solution (Sigma Aldrich) and the presence of CD14/CD206 double-positive cells was analyzed on a FACS Verse (BD Biosciences, San Diego, CA, USA). Dead cells were excluded by 7AAD-staining (BD Biosciences, 559925, 1:1000) and the presence of dendritic cells was excluded by CD1a staining. Antibodies used were: CD14-FITC (555397, 1:10), CD206-APC (550889, 1:20), and CD1a-PE (555807, 1:10), all from BD Biosciences.

### Lipid Staining of Foam Cell Macrophages

For Oil Red O staining, cultured monocytes were washed and fixed in 4% paraformaldehyde. Oil Red O (Sigma Aldrich) in 60% isopropanol was added to the culture dish and cells were stained for 1 h in room temperature. Excess Oil Red O solution was washed away with water followed by isopropanol and pictures were taken under light microscope. For staining of tissue, 5 μm fresh frozen tissue sections were stained for 20 min using an Oil Red O solution in 67% isopropanol. Excess staining was removed as described above, slides were counterstained with hematoxylin and mounted in Aqua pertex (Histolab, Gothenburg, Sweden).

### Statistics

GraphPad Prism software was used for statistical analyses. Pair-wise comparisons were made using a paired, two-tailed Student’s *t*-test. For multiple comparisons, one-way ANOVA was used followed by Dunnett’s correction for multiple comparisons. *P*-values<0.05 were considered significant.

## RESULTS

### Foamy Macrophages are Commonly Found within pRCC Tumors

**Figure 1:**
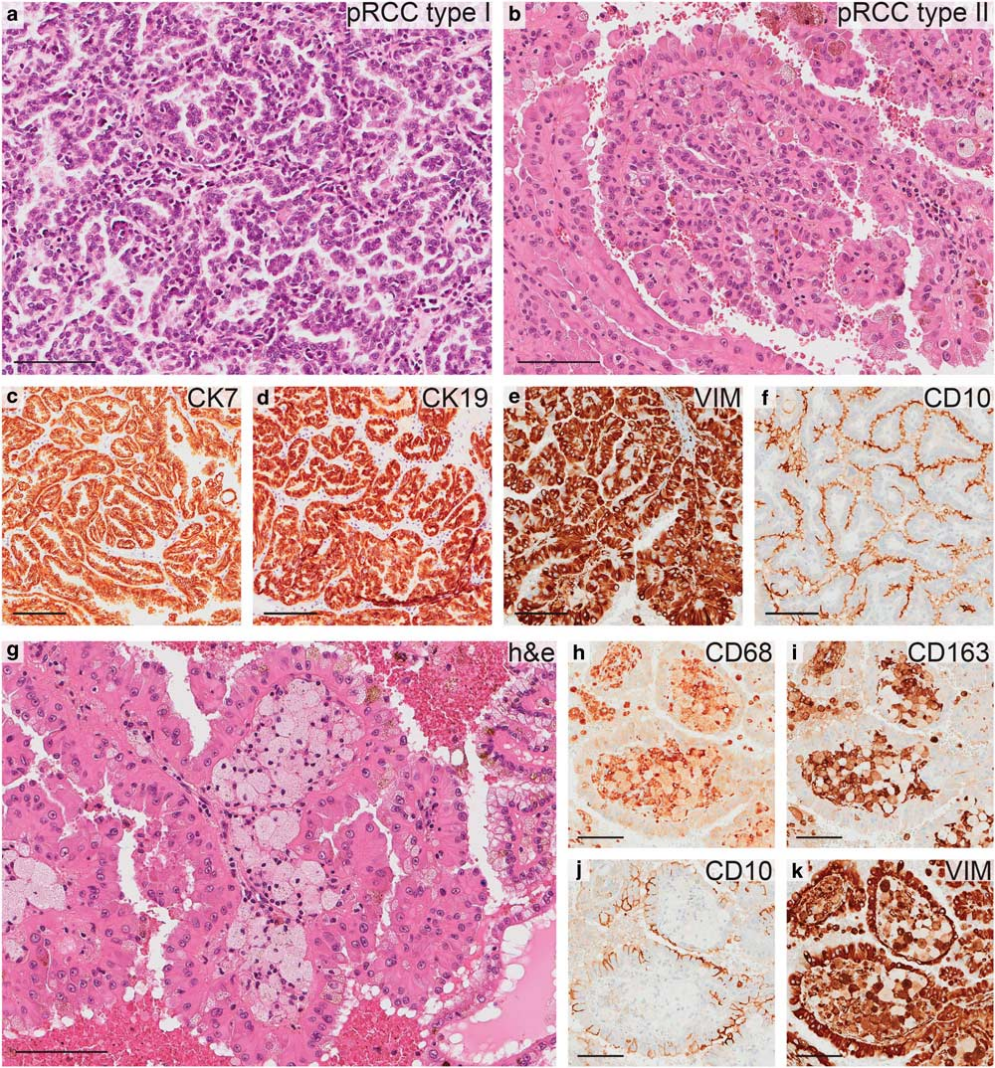
Histology of papillary RCC. Hematoxylin and eosin staining of papillary RCC samples showing the characteristic growth pattern of type 1 (**a**) and type 2 (**b**) pRCC tumors. Immunohistochemical staining showing an example of a pRCC tumor positive for cytokeratins 7 and 19 (CK7, CK19) (**c**, **d**), vimentin (VIM) (**e**), and CD10 (**f**). Accumulation of enlarged macrophages is commonly found in the papillary stalk (**g**). These macrophages stain positive for CD68 (**h**) and CD163 (**i**). The surrounding tumor cells are visualized by CD10 staining (**j**). Both macrophages and immune cells are positive for vimentin (**k**). Scale bars=100 μm.

The characteristic papillary growth pattern of pRCC is found in both type 1 and type 2 tumors. Histopathologically, type 1 pRCCs are recognized by the relatively smaller tumor cells, usually with low nuclear grade and amphophilic cytoplasm (Figure 1a). The more aggressive type 2 tumor cells are larger, eosinophilic, and display nuclear pseudostratification (Figure 1b). pRCCs of both subtypes are identified immunohistochemically by their expression of cytokeratins 7 and 19 as well as vimentin,^12^ as exemplified in Figures 1c–e. Their proximal tubule origin also renders them positive for CD10^13^ (Figure 1f). In Figure 1g, a pRCC tumor section containing the distinctive accumulation of enlarged foamy macrophages is shown. In order to evaluate the prevalence and phenotype of foamy macrophages in pRCC tumors, retrospective analysis was performed on histological slides obtained from 140 individual cases of pRCC. In this material, presence of aggregated foamy macrophages was noted in 82% of all tumors (Table 1). Immunohistochemical staining for CD68 and CD163 was performed on a tissue micro array (TMA) containing 51 pRCC tumors (clinical and histopathological data are summarized in Supplementary Table 1). In all cases, the foamy macrophages stained positive for CD68 (exemplified in Figure 1h), and also for CD163 (Figure 1i), indicating an anti-inflammatory M2-phenotype.^14^ As expected, the surrounding pRCC tumor cells were positive for CD10 (Figure 1j) and both cell types displayed an expression of vimentin (Figure 1k). In accordance with previous publications suggesting a prognostic value of the presence of macrophages,^15^ tumors lacking foamy macrophages tended to be larger and of higher grade (Table 2).

Of note, 20% of the pRCC samples contained areas with features of both type 1 and type 2. In this group, the percentage of foamy macrophage containing tumors was 89%.

According to these results, foamy macrophages of M2 phenotype are present in the majority of pRCC tumors, indicating that the attraction and differentiation of macrophages into foam cells seem to be an intrinsic property of pRCC tumors.

### Isolation and Culture of Human Primary pRCC Cells

**Figure 2:**
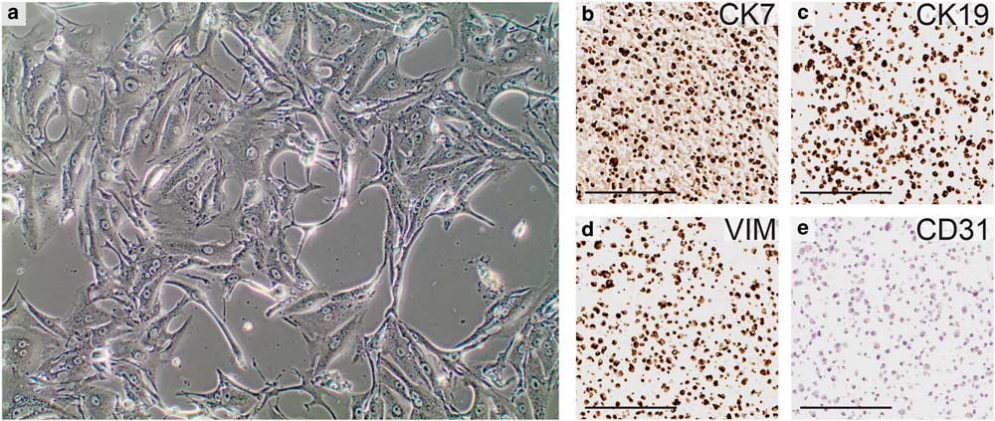
Characterization of cultured primary pRCC cells. Phase contrast microscopy image of isolated human primary pRCC cells in culture at × 10 magnification (**a**). Immunohistochemical stainings of formalin-fixed and paraffin-embedded cultured cells show positive staining for cytokeratin 7 (CK7) (**b**), cytokeratin 19 (CK19) (**c**), and vimentin (VIM) (**d**). Staining for the endothelial marker CD31 is negative (**e**). These results confirm the pRCC origin of the cultured cells. Scale bars=100 μm.

To our knowledge, there are few, if any, established cell lines with a confirmed pRCC origin available. Therefore, in order to study the interaction between pRCC cells and monocytes *in vitro*, we established cultures from pRCC tumor samples derived from patient nephrectomies. The morphology of these cells is shown in Figure 2a. The pRCC origin of the cultured cells was confirmed by immunohistochemical stainings for the pRCC markers CK7, CK19, and vimentin (Figures 2b–d). The cultured cells were negative for CD31, excluding the potential presence of contaminating endothelial cells (Figure 2e).

### Cultured Primary Human pRCC Cells Secrete Factors that Attract Monocytes

**Figure 3:**
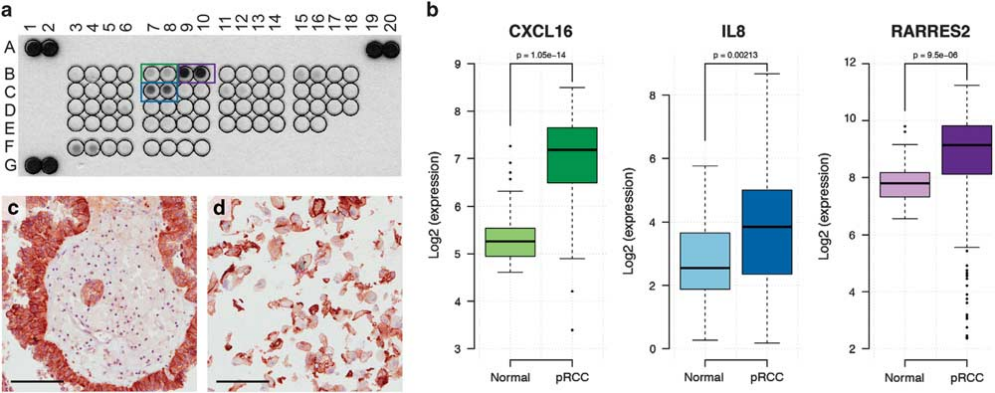
Chemokines produced by human pRCC cells. Human chemokine proteome profiler antibody array analysis of conditioned medium from cultured human primary pRCC cells (**a**). The three strongest signals are from CXCL16 (B7, B8); chemerin (B9, B10), and IL-8 (C7, C8). The signal at F3, F4 is fibrinogen (sample control). The upregulation of chemokines in pRCC tissue compared to normal kidney samples is confirmed on RNA level (**b**). Boxplots show relative mRNA gene transcript levels of *CXCL16*, *RARRES2*, and *IL-8* in TCGA RNAseq data comprising 32 normal kidney tissue samples and 234 pRCCs. *P*-values were calculated using Wilcoxon’s test. The expression of CXCL16 protein in pRCC tumor cells is demonstrated by immunohistochemical staining of pRCC tumor tissue (**c**) and in cultured cells (**d**). Note the plasma membrane localization. Scale bars=100 μm.

The presence of large amounts of macrophages within pRCC tumors prompted us to hypothesize that the pRCC tumor cells secrete factors attracting monocytes, that differentiate into macrophages within the tumor tissue. To determine what factors are released from pRCC tumor cells, conditioned medium from the primary cultures of human pRCC cells was collected and analyzed using a proteome profiler chemokine array. Several chemokines could be detected and the strongest signals were retrieved from CXCL16, chemerin (RARRES2) and IL-8 (Figure 3a). Importantly, increased expression of these genes in pRCC tumors compared to normal kidney tissue was confirmed in RNA sequencing data available from the TCGA (Figure 3b). Both IL-8 and chemerin are secreted factors, making immunohistochemical staining for these chemokines difficult to perform. In contrast, CXCL16 exists in both membrane-bound and secreted forms.^16^ Immunohistochemical staining of pRCC tissue (Figure 3c) and cultured pRCC cells (Figure 3d) for CXCL16 confirmed the expression of this protein in the tumor cells, displaying a membranous localization.

**Figure 4:**
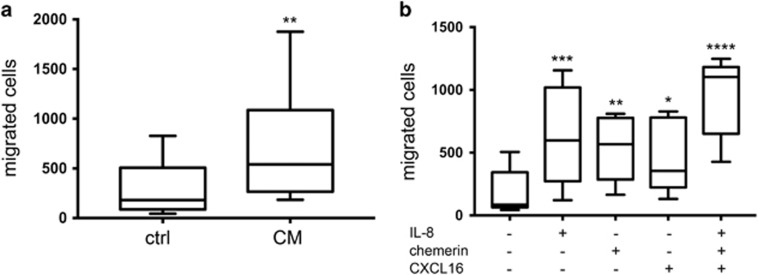
pRCC cell secreted factors attract human monocytes. Results from migration assay showing the number of human monocytes that after 24 h have migrated toward conditioned medium (CM) from pRCC cultures (**a**, *n*=7; Student’s *t*-test), or the indicated recombinant chemokines separately or combined (**b**, *n*=5; ANOVA), compared to control medium. Asterisks indicate the significance of the results compared to control medium where *P<0.05; **P<0.01; ***P<0.001; and ****P<0.0001.

To test whether conditioned medium from pRCC cultures, as well as the individual factors CXCL16, IL-8, or chemerin could attract monocytes, migration assays were performed. Monocytes were isolated from human PBMCs and seeded into the upper well of a Boyden chamber. pRCC-conditioned medium, CXCL16, IL-8, chemerin or control medium was added to the lower compartment. After 24 h, the number of migrated monocytes was determined by flow cytometry. As shown in Figure 4a, there was a significant increase in the number of migrated monocytes toward pRCC-conditioned medium compared to control medium. The chemokines identified in the proteome profiler chemokine array also attracted human monocytes in a similar range (Figure 4b). The combination of all three chemokines resulted in the largest number of migrated monocytes.

### Conditioned Medium from Human Primary pRCC Cells Induces a Phenotypic Switch and Lipid Accumulation in Human Monocytes

**Figure 5:**
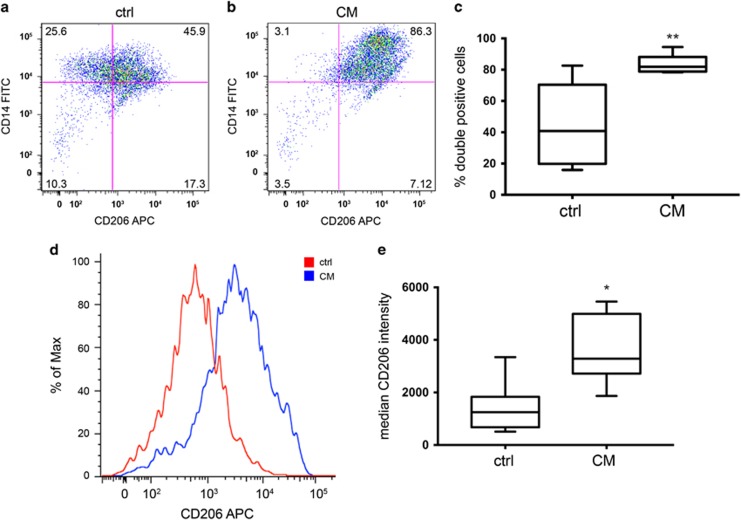
Conditioned medium from pRCC induces a phenotypic shift in monocytes. After 7 days of culture in the presence of pRCC-conditioned medium, the percentage of CD14/CD206 double-positive cells was increased (**a**) compared to control medium (**b**). Scatter plots from a representative experiment are shown. In (**c**), the percentage of double-positive cells summarized from eight experiments is shown. (**d**). Representative histogram demonstrating the increase in CD206 intensity of CD14 positive cells after culture in pRCC-conditioned medium (blue) compared to control medium (red). In (**e**), the results from eight experiments are summarized. Student’s *t*-test; asterisks indicate the significance of the results compared to control medium where *P<0.05; **P<0.01; ***P<0.001; and ****P<0.0001.

In order to assess how factors secreted from the cultured human pRCC cells affect the phenotype of human monocytes, isolated monocytes were cultured for an extended period of time in the presence of pRCC-conditioned medium. After 7 days, the monocytes were harvested and their phenotype was analyzed by flow cytometry. As depicted in Figure 5, growth in the presence of the pRCC-conditioned medium resulted in a shift of phenotype, skewed toward M2 macrophages. Cells positive for the monocyte/macrophage marker CD14 became increasingly positive for the M2 marker CD206. Both the percentage of CD14/CD206 double-positive cells (Figures 5a–c), as well as the CD206 staining intensity of the CD14 positive cells (Figures 5d and e) increased significantly. All cells were negative for the dendritic cell marker CD1a (data not shown).

**Figure 6:**
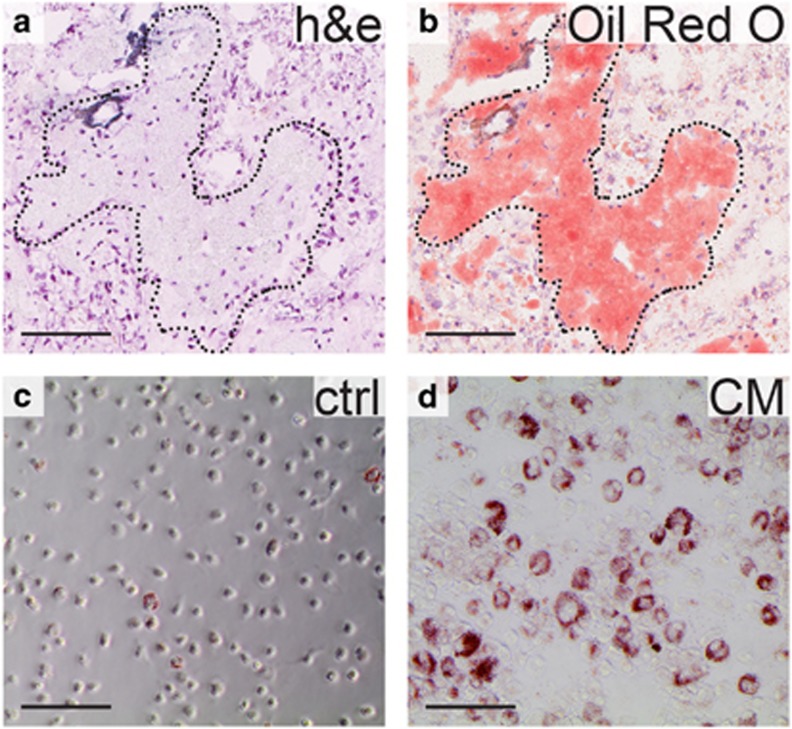
pRCC-conditioned medium promotes lipid accumulation in cultured monocytes. Hematoxylin/eosin (**a**) and Oil Red O (**b**) staining of fresh-frozen pRCC tissue demonstrating the lipid content of the tumor-associated macrophages, outlined with the dotted line. Culture of isolated human monocytes for 14 days in pRCC-conditioned medium resulted in enlarged, lipid-laden cells. Oil Red O staining of cultured monocytes in control medium (**c**) or pRCC-conditioned medium (**d**). Also, note the difference in size. Scale bars=100 μm.

Furthermore, we observed that continued culture of monocytes in the presence of pRCC-conditioned medium resulted in an altered morphology with an increase in size (compare Figures 6c and d). The foam cells/TAMs commonly seen within pRCC tumors are enlarged in a similar way as the foam cells found in atherosclerotic plaques. The content of these cells has not been well investigated in pRCC tissue, however. Oil Red O staining of fresh-frozen pRCC tumor tissue containing macrophages demonstrated that the enlarged macrophages indeed contained an accumulation of cytoplasmic lipids (Figures 6a and b). Staining of monocytes cultured for 10 days in the presence of pRCC-conditioned medium also revealed an increased cytoplasmic lipid content compared to monocytes cultured in control medium (Figures 6c and d), suggesting that factors secreted by pRCC tumor cells induce the lipid accumulation seen in the pRCC tumor-associated foamy macrophages.

## DISCUSSION

A papillary growth pattern combined with focal aggregation of foam cell macrophages inside the papillae constitute a unique and pathognomonic histology for the identification of pRCC.

In this report, we show that foamy macrophages are found in over 80% of pRCC tumors, a figure slightly higher than what has previously been reported.

From other malignancies, we have learnt that factors produced by TAMs influence key processes of the adjacent tumor cells such as migration, invasion, and proliferation. In ccRCC, TAMs of M2 phenotype have been shown to affect the function and phenotype of infiltrating T cells, and the immune cell composition within ccRCC tumors has been linked to disease stage.^17, 18^ The situation in pRCC is not as well described. A few descriptive studies have presented data regarding the frequency of pRCC macrophage accumulation and macrophage type. According to Behnes *et al*,^19^ focal accumulation of TAMs is equally abundant in type 1 and type 2 pRCC, but the more aggressive type 2 tumors contain more CD163 positive macrophages of the M2 type. On the contrary, in another study the presence of TAMs was reported to be associated with lower tumor grade and absence of vascular invasion in pRCC.^15^ The discrepancies between these studies could probably at least partly be due to the focal appearance of TAMs in pRCC, hence, the extent of tumor sampling will influence the results. According to our histological and experimental data, the pRCC-associated foamy macrophages are of the tumor promoting M2 phenotype. Further studies need to be performed in order to establish how these TAMs affect the tumor microenvironment and the phenotype of other tumor infiltrating myeloid cells.

In some pRCC tumors, areas of both type 1 and type 2 morphology can be found. In our patient cohort, 20% of the analyzed tumors contained both subtypes. Interestingly, this group of pRCC tumors displayed the highest percentage of focal macrophage accumulation (89% compared to 82% for type 1 and 77% for type 2). Tumors containing both subtypes are usually classified into type 1 or type 2, either based on the predominant subtype, or using the principle that tumors containing any type 2 elements are classified as type 2. However, no established guidelines exist regarding which principle to use, and in order not to obscure scientifically relevant information, we chose to keep this biphasic category as a separate group. Recent comprehensive molecular characterization of pRCC^1, 4^ has given new insights into pRCC taxonomy. Data indicate that both type 1 and type 2 pRCC contain further subtypes with its own molecular and genetic profiles. Again, accounting for both separate and compound phenotypes might give more information in this rapidly evolving field.

It is interesting to note that macrophages found within pRCC tumors of all subtypes display a foamy phenotype, indicating that the signaling contributing to the development of the foamy phenotype is an intrinsic property of pRCC tumors. A possible exception might be some high-grade tumors with lower degree of differentiation, which might imply that these tumors have lost their original pRCC chemokine profile that initially caused accumulation of foamy macrophages.

Despite the established phenomenon of foam cell accumulation in pRCC, very little is known regarding the cellular processes behind this histology, or what effects tumor and myeloid cells possibly exert on each other.

To our knowledge, there are few, if any, established cell lines of pRCC origin available. Primary culture of pRCC cells is rarely, if ever, performed on a regular basis, probably due to the lack of access to fresh pRCC material. The number of publications addressing functional aspects of pRCC is therefore low. In this study, we succeeded in establishing cultures from pRCC tissue dissected from patient nephrectomies, thereby generating the necessary tools to study how pRCC tumor cells influence human myeloid cells.

By analyzing the chemokines secreted from primary pRCC cells, we identified several factors present in the conditioned pRCC medium. Interestingly, we could not detect the classical monocyte attracting chemokine monocyte chemoattractant protein 1 (MCP1 or CCL2), that is overexpressed in several tumor types and correlate to TAM infiltration. Instead, high levels of CXCL16, IL-8 and chemerin were found. Previously, these factors have individually been shown to attract human blood monocytes and macrophages in various settings.^8, 20, 21^ The combination of these three factors has however not previously been identified to be driving TAM recruitment into tumor tissue. In this study, we show that CXCL16, IL-8, and chemerin (all present in conditioned medium from pRCC cell cultures) attract primary human blood monocytes, both individually and in combination.

IL-8 was first identified as a chemotactic factor activating neutrophils, and it was thought to be produced by activated macrophages and monocytes. Later it was shown that other cell types could produce IL-8 and that it is upregulated in several types of malignancies, contributing to processes such as proliferation, angiogenesis, invasiveness, and metastasis.^22^ IL-8 has recently been shown to be upregulated and correlated to metastasis and epithelial–mesenchymal transition also in a RCC cell line and RCC tissue, probably of clear cell origin.^23^ However, to our knowledge, our study is the first to identify IL-8 as a factor being produced by pRCC cells.

Like IL-8, increased expression of CXCL16 has been observed in other cancer forms and correlates to metastatic potential and worse prognosis in several cancer types.^8, 24, 25^ In renal cancer on the contrary, the promoter region of *CXCL16* has been shown to be methylated in some cases,^26^ and high expression of CXCL16 has instead been linked to longer survival time and inhibited cancer cell migration.^27^ However, the distinction between different types of RCC in these reports are vague, and none of the cell lines used in the functional assays were of papillary origin, hence the results are difficult to interpret in this context.

Initially isolated from psoriatic skin lesions, chemerin is mainly expressed by adipose tissue, liver, and placenta, but also in pancreas, kidney, and the gastrointestinal tract.^28, 29^ This factor has however not been shown to be expressed by any type of RCC previously. Chemerin is an adipokine and has been shown to play an important role in the metabolic syndrome linking inflammation of adipose tissue with insulin resistance and body fat accumulation. The correlation to lipid metabolism is interesting given the foam cell macrophage phenotype seen in pRCC tumors. A potential involvement of chemerin in pRCC macrophage lipid accumulation remains to be investigated.

To generalize our findings, we analyzed RNAseq gene expression data of 234 pRCC cases and 32 normal kidney tissue samples deposited in the TCGA data base and transcript levels for *IL-8*, *CXCL16*, and *RARRES2* were all found to be upregulated in pRCC cases. These factors, alone or in combination, were able to attract primary human monocytes in a migration assay, as was conditioned medium from primary human pRCC cultures. The conditioned medium from pRCC cells furthermore induced a switch, where monocytes developed into M2-like macrophages, defined by a CD14^+^CD206^+^CD1a^−^ surface marker phenotype.

The pRCC macrophages are morphologically similar to the lipid-laden foam cells found in atherosclerotic plaques. However, the contents of pRCC foam cell macrophages has not been formally characterized. In this study, we therefore performed Oil Red O staining of pRCC tissue macrophages, confirming a lipid content. Long-term culture of monocytes in pRCC-conditioned medium induced a similar enlarged, lipid-filled morphology of macrophages *in vitro*.

The potential interplay between the aggregated macrophages and the pRCC tumor cells is an interesting area where many questions remain to be answered. Based on our results, we propose that pRCCs and myeloid cells have a unique crosstalk. The ability to attract monocytes and differentiate them into foam cells of M2 phenotype seems to be an intrinsic property of pRCC tumors. How the presence of foamy TAMs in pRCC affects tumor biology remains to be elucidated. Potentially, novel therapies that block myeloid cell recruitment and differentiation, such as CSF1 inhibitors, might have a role in the treatment of pRCC patients.

## Figures and Tables

**Table 1:** Summary of clinical and histopathological data of 140 analyzed pRCC cases

	Total	Type 1	Type 2	Types 1 and 2
*n* (%)	140 (100)	68 (49)	44 (31)	28 (20)
Fuhrman grade 1	6 (4)	5 (7)	1 (2)	0 (0)
2	72 (51)	49 (72)	15 (34)	8 (29)
3	44 (31)	10 (15)	16 (36)	18 (64)
4	18 (13)	4 (6)	12 (27)	2 (7)
WHO/ISUP grade 1	33 (24)	29 (43)	3 (7)	1 (4)
2	49 (35)	27 (40)	14 (32)	8 (29)
3	42 (30)	8 (12)	17 (39)	17 (61)
4	16 (11)	4 (6)	10 (23)	2 (7)
Male	110 (79)	54 (79)	34 (77)	22 (79)
Female	30 (21)	14 (21)	10 (23)	6 (21)
Macrophage presence	115 (82)	56 (82)	34 (77)	25 (89)
Mean age	66	64	70	67
Mean size (mm)	60	52	73	63

**Table 2:** Patient data grouped according to tumor type and macrophage (MΦ) presence

	All		Type 1		Type 2		Type 1 & 2	
	w MΦ	wo MΦ	w MΦ	wo MΦ	w MΦ	wo MΦ	w MΦ	wo MΦ
*n* (%)	115 (82)	25 (18)	56 (82)	12 (18)	34 (77)	10 (23)	25 (89)	3 (11)
								
*Fuhrman grade*								
1	2 (2)	4 (16)	1 (2)	4 (33)	1 (3)	0 (0)	0 (0)	0 (0)
2	65 (57)	7 (28)	45 (80)	4 (33)	13 (38)	2 (20)	7 (28)	1 (33)
3	37 (32)	7 (28)	8 (14)	2 (17)	13 (38)	3 (30)	16 (64)	2 (67)
4	11 (10)	7 (28)	2 (4)	2 (17)	7 (21)	5 (50)	2 (8)	0 (0)
								
*WHO/ISUP grade*								
1	28 (24)	5 (20)	24 (43)	5 (42)	3 (9)	0 (0)	1 (4)	0 (0)
2	42 (37)	7 (28)	24 (43)	3 (25)	11 (32)	3 (30)	7 (28)	1 (33)
3	34 (30)	8 (32)	6 (11)	2 (17)	13 (38)	4 (40)	15 (60)	2 (67)
4	11 (10)	5 (20)	2 (4)	2 (17)	7 (21)	3 (30)	2 (8)	0 (0)
Male	91 (79)	19 (76)	45 (80)	9 (75)	27 (79)	7 (70)	19 (76)	3 (100)
Female	24 (21)	6 (24)	11 (20)	3 (25)	7 (21)	3 (30)	6 (24)	0 (0)
Mean age	67	66	65	60	69	72	68	66
Mean size (mm)	57	79	50	64	65	104	61	77
