# Supplementary material for: Papillary renal cell carcinoma-derived chemerin, IL-8, and CXCL16 promote monocyte recruitment and differentiation into foam-cell macrophages
Source: Lab Invest. 2017 Jul 31;97(11):1296–305. doi: 10.1038/labinvest.2017.78 (PMC5668481; doi:10.1038/labinvest.2017.78)
Supplement: Supplementary Table S1 [file labinvest201778x1.pdf]

Supplementary Table 1. Summary of clinical and histopathological data of the 51 pRCC cases included in the TMA.

		Total	Type 1	Type 2	Type 1 & 2
n (%)		51 (100)	28 (55)	15 (29)	8 (16)
Fuhrman grade	1	4 (8)	4 (14)	0 (0)	0 (0)
	2	27 (53)	20 (71)	5 (33)	2 (25)
	3	16 (31)	4 (14)	6 (40)	6 (75)
	4	4 (8)	0 (0)	4 (27)	0 (0)
WHO/ISUP grade	1	13 (26)	12 (43)	0 (0)	1 (13)
	2	20 (39)	13 (46)	6 (40)	1 (13)
	3	15 (29)	3 (11)	6 (40)	6 (75)
	4	3 (6)	0 (0)	3 (20)	0 (0)
Male		44 (86)	23 (82)	14 (93)	7 (88)
Female		7 (14)	5 (18)	1 (7)	1 (13)
Macrophage presence		40 (78)	24 (83)	10 (67)	6 (86)
Mean age		65	63	67	74
Mean size (mm)		58	50	69	66
